# Supplementary material for: Persistent spin textures, altermagnetism and charge-to-spin conversion in metallic chiral crystals TM3X6
Source: Npj Spintron. 2025 Nov 3;3(1):46. doi: 10.1038/s44306-025-00109-9 (PMC12583138; doi:10.1038/s44306-025-00109-9)
Supplement: Supplementary file 1 — Supplementary Information [file 44306_2025_109_MOESM1_ESM.pdf]

Supplementary Information for

Persistent spin textures, altermagnetism and charge-to-spin conversion in metallic chiral crystals TM_3X_6

Karma Tenzin,[1] Berkay Kilic,[1] Raghottam M Sattigeri,[2] Zhiren He,[3] Chao Chen Ye,[1] Marcio Costa,[4] Marco Buongiorno Nardelli,[3] Carmine Autieri,[5,6] and Jagoda Sławińska[1]

[1]*Zernike Institute for Advanced Materials, University of Groningen, Nijenborgh 3, 9747 AG Groningen, The Netherlands*

[2]*Physics Department, Università degli Studi di Milano, Via Celoria 16, 20133 Milan, Italy*

[3]*Department of Physics, University of North Texas, Denton, TX 76203, USA*

[4]*Instituto de Física, Universidade Federal Fluminense, 24210-346, Niterói RJ, Brazil*

[5]*International Research Centre MagTop, Institute of Physics, Polish Academy of Sciences, Aleja Lotników 32/46, PL-02668 Warsaw, Poland*

[6]*SPIN-CNR, c/o Università di Salerno, IT-84084 Fisciano (SA), Italy*

Contents

S1: Relativistic spin-resolved electronic structures of $NiTa_3S_6$ 2

S2: Relativistic spin-resolved electronic structures of $NiNb_3S_6$ 4

S3: Projected density of states and resistivity 6

S4: Altermagnetism and weak ferromagnetism in $NiNb_3S_6$ 7

S5: Rashba-Edelstein effect and spin Hall effect in $NiNb_3S_6$ 8

S6: Charge-to-spin conversion in $NiTa_3S_6$ and $NiNb_3S_6$ vs reference materials 10

S1: Relativistic spin-resolved electronic structures of $NiTa_3S_6$

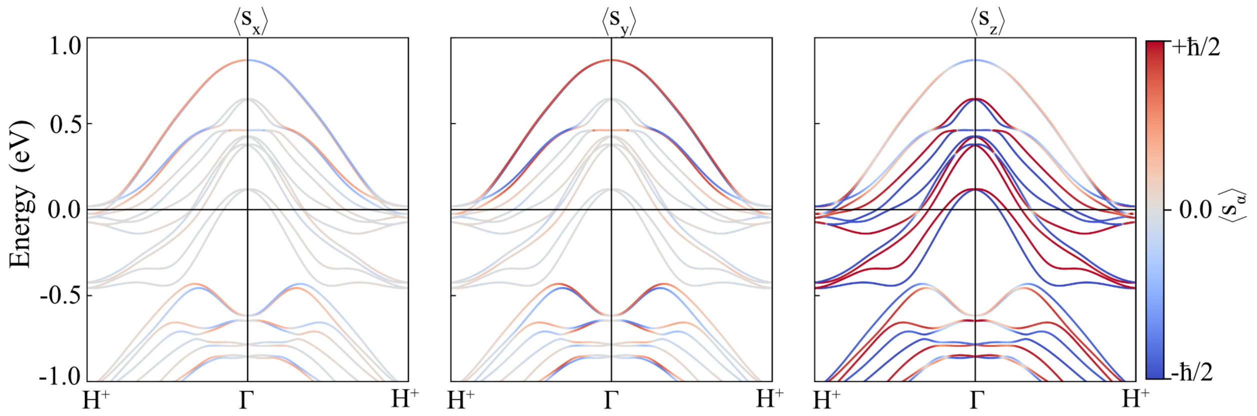

Supplementary figure 1: Spin-resolved band structures calculated for **nonmagnetic** $NiTa_3S_6$.

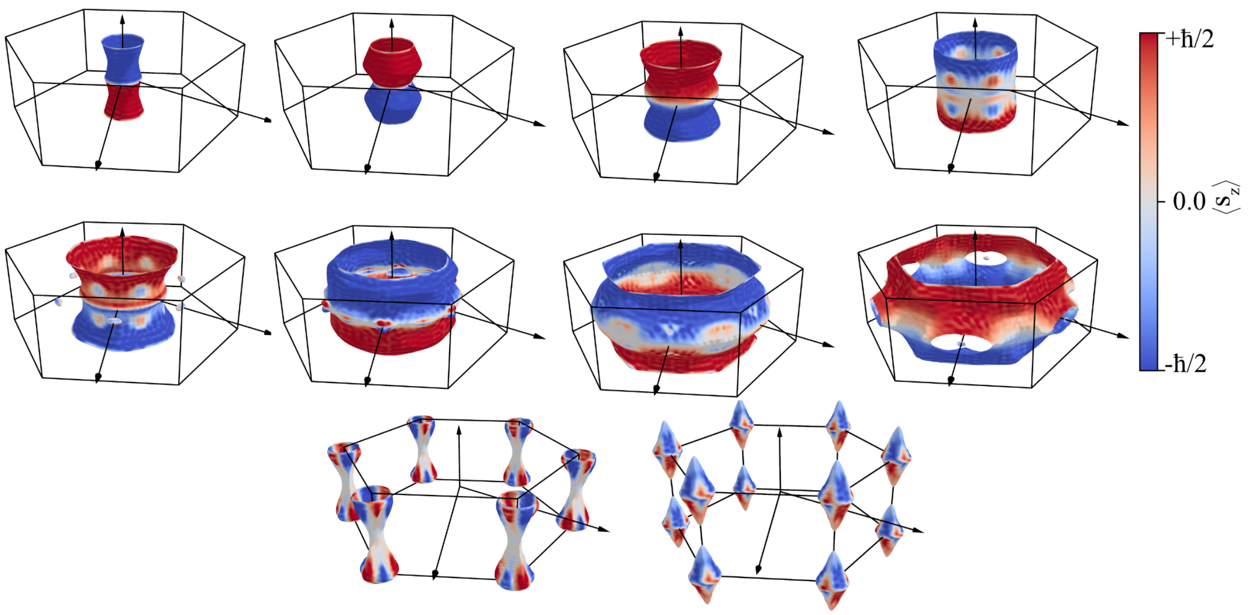

Supplementary figure 2: Fermi surfaces of **nonmagnetic** $NiTa_3S_6$. The surfaces shown here correspond to the true Fermi level $E_F = 0.0$. The color represents the S_z component of the spin texture.

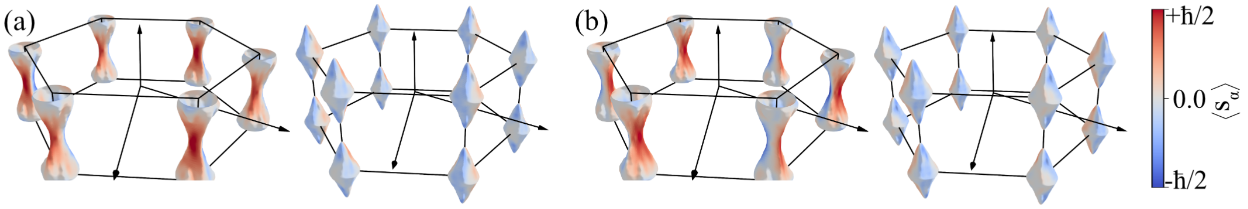

Supplementary figure 3: Fermi surfaces of **nonmagnetic** $NiTa_3S_6$. The surfaces shown here correspond to the true Fermi level $E_F = 0.0$. The color represents the S_x (a) and S_y (b) component of the spin texture.

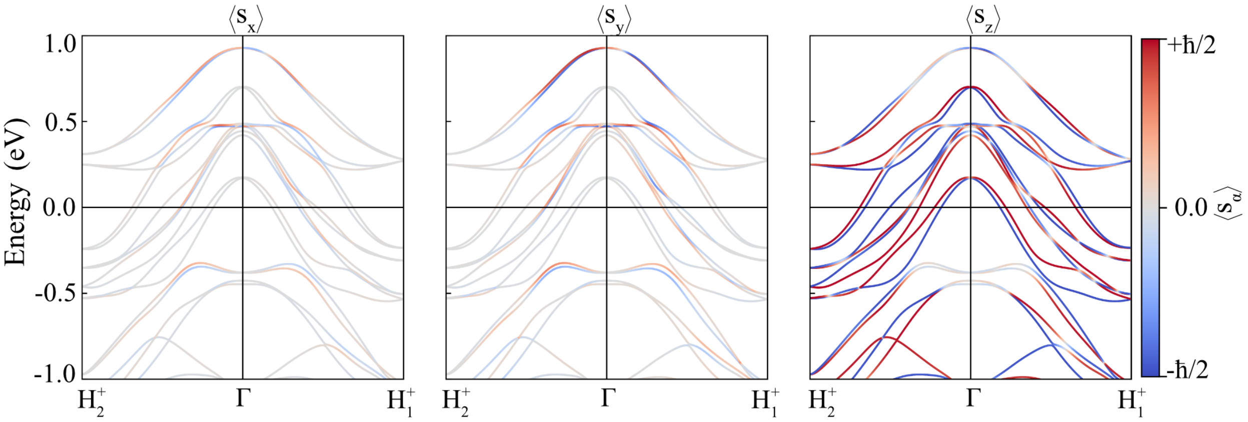

Supplementary figure 4: Spin-resolved band structures calculated for **altermagnetic** $NiTa_3S_6$ assuming the Néel vector along the [001] direction. SOC was included self-consistently at the DFT level.

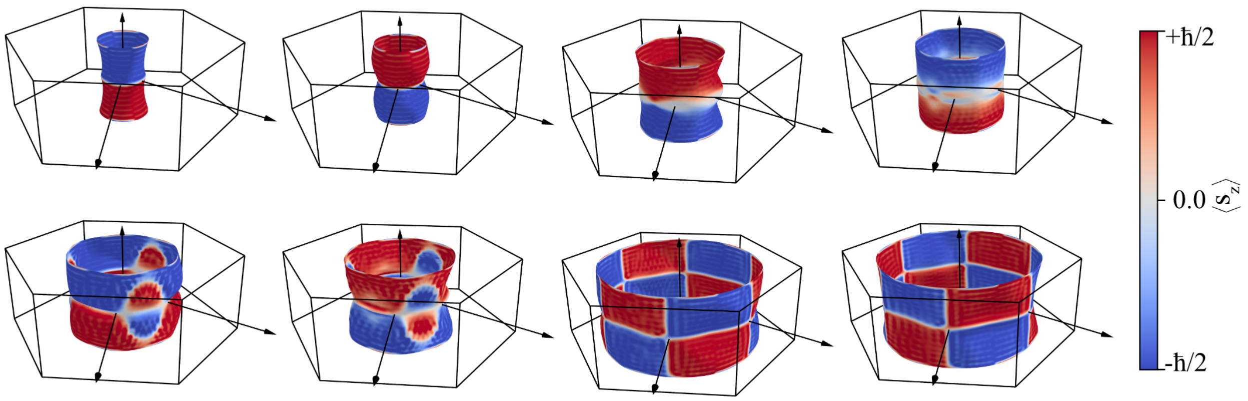

Supplementary figure 5: Fermi surfaces of altermagnetic $NiTa_3S_6$. The surfaces correspond to the true Fermi level $E_F = 0.0$. The color represents the spin polarization along the direction of the Néel vector [001].

S2: Relativistic spin-resolved electronic structures of $NiNb_3S_6$

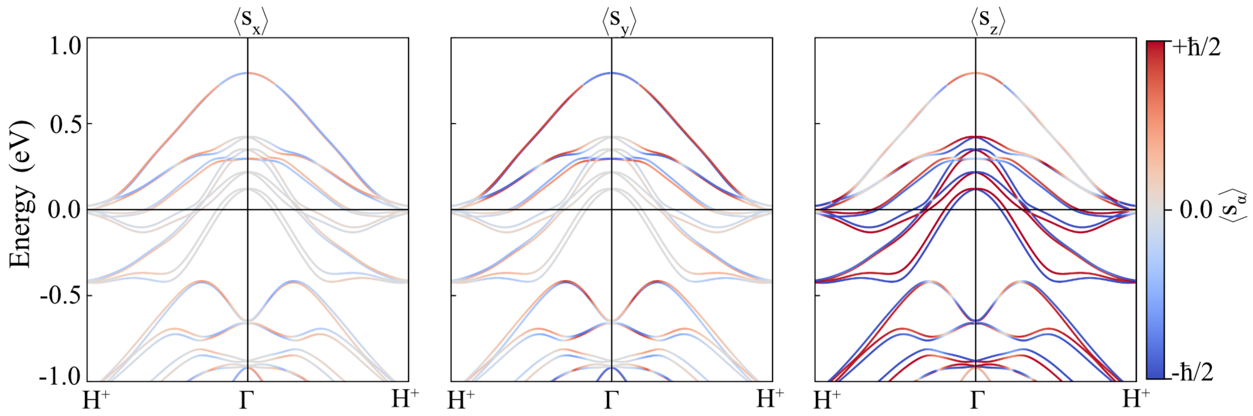

Supplementary figure 6: Spin-resolved band structures calculated for **nonmagnetic** $NiNb_3S_6$.

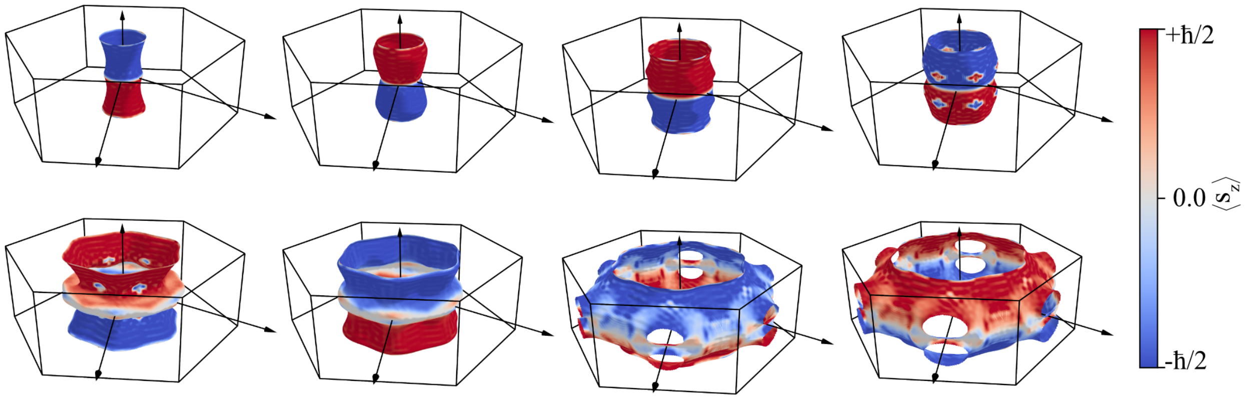

Supplementary figure 7: Fermi surfaces calculated for **nonmagnetic** $NiNb_3S_6$. The surfaces shown here correspond to the true Fermi level $E_F = 0.0$. The color represents the S_z component of spin texture.

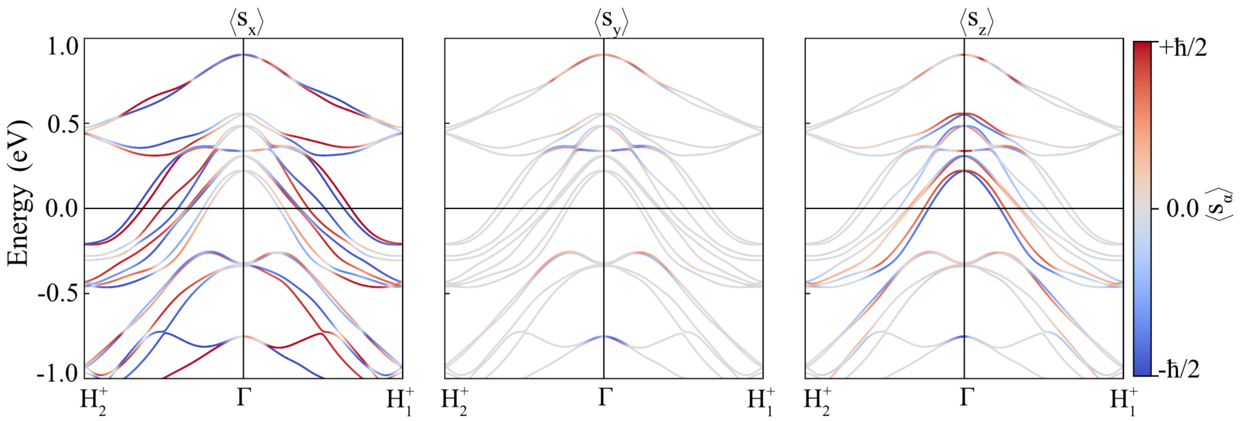

Supplementary figure 8: Spin-resolved band structures calculated for **altermagnetic** $NiNb_3S_6$.

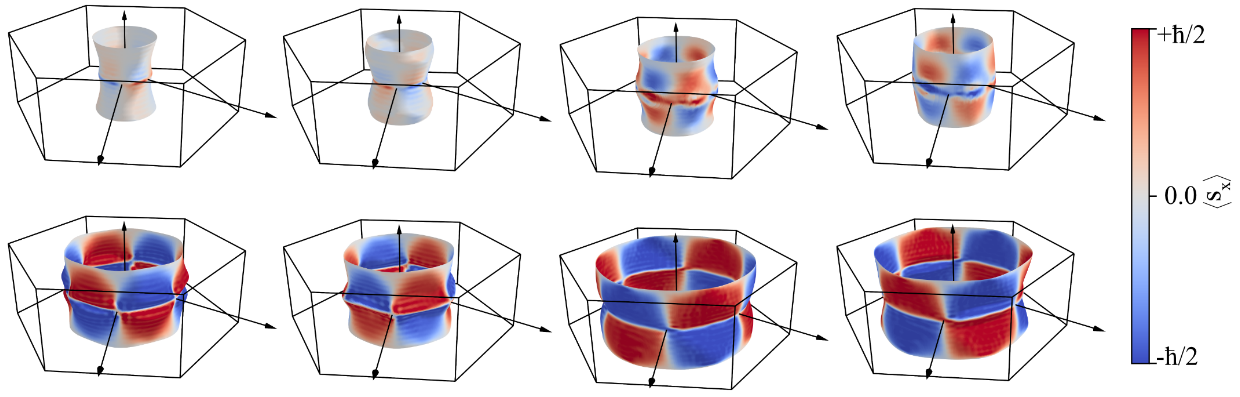

Supplementary figure 9: Fermi surfaces of altermagnetic $NiNb_3S_6$. The surfaces correspond to the true Fermi level $E_F = 0.0$. The color represents the spin texture along the direction of the Néel vector [100].

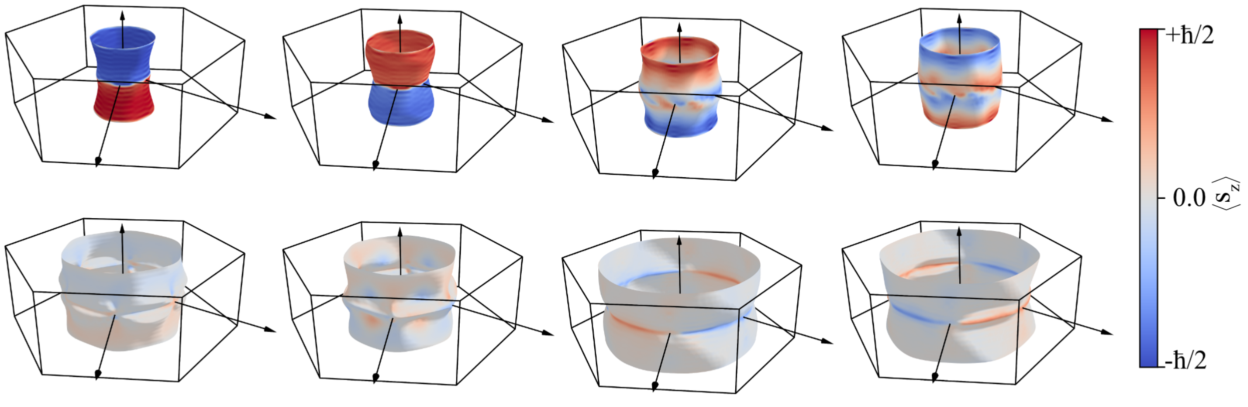

Supplementary figure 10: Same as Supplementary figure 8 but with the color representing S_z projection.

S3: Projected density of states and resistivity

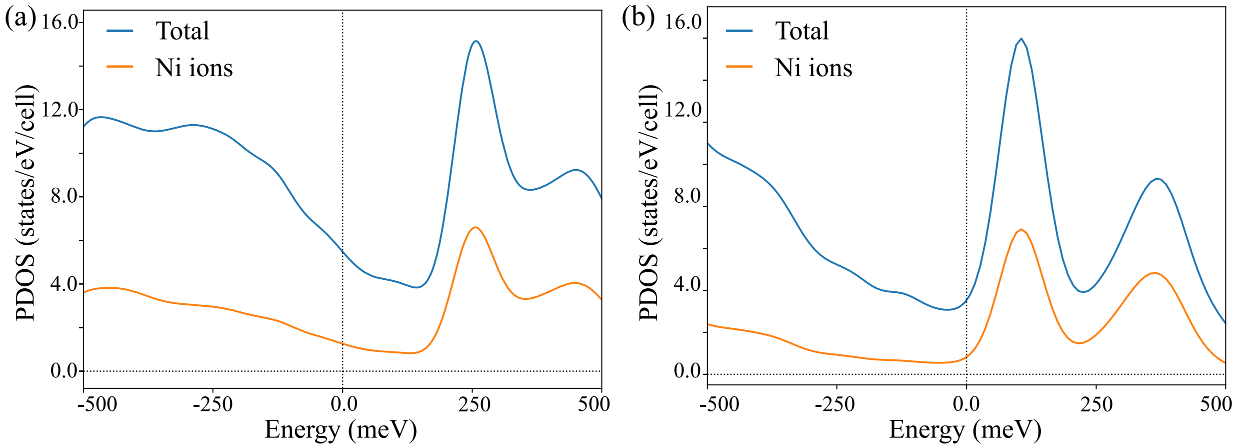

Supplementary figure 11: Projected density of states of altermagnetic $NiTa_3S_6$ (a) and $NiNb_3S_6$ (b).

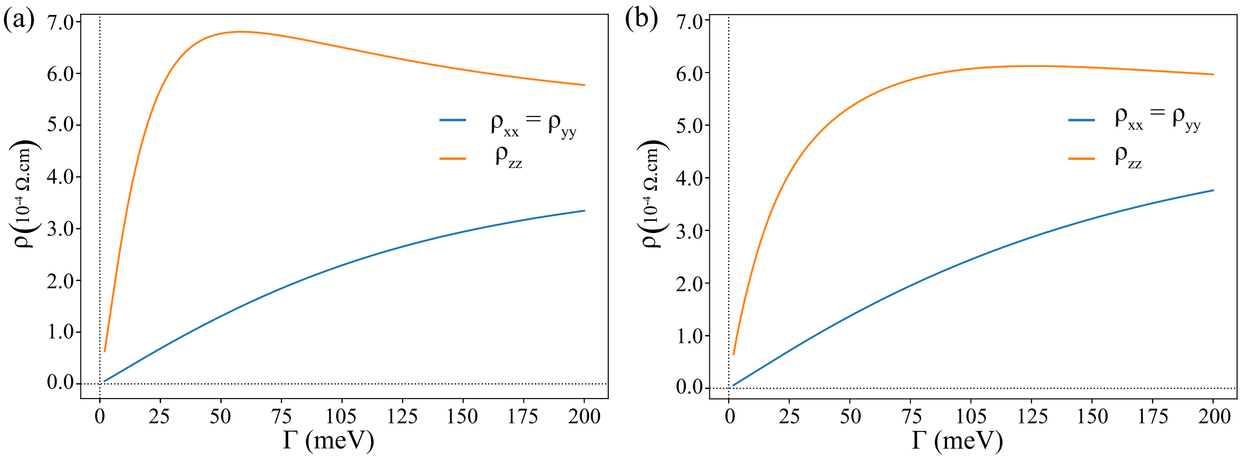

Supplementary figure 12: Calculated resistivity as a function of disorder parameter Γ for altermagnetic $NiTa_3S_6$ (a) and $NiNb_3S_6$ (b). We set the Néel vector along [001] direction and Hubbard $U = 0$ eV for $NiTa_3S_6$, and Néel vector along [100] direction and $U = 1.0$ eV for $NiNb_3S_6$.

S4: Altermagnetism and weak ferromagnetism in $NiNb_3S_6$

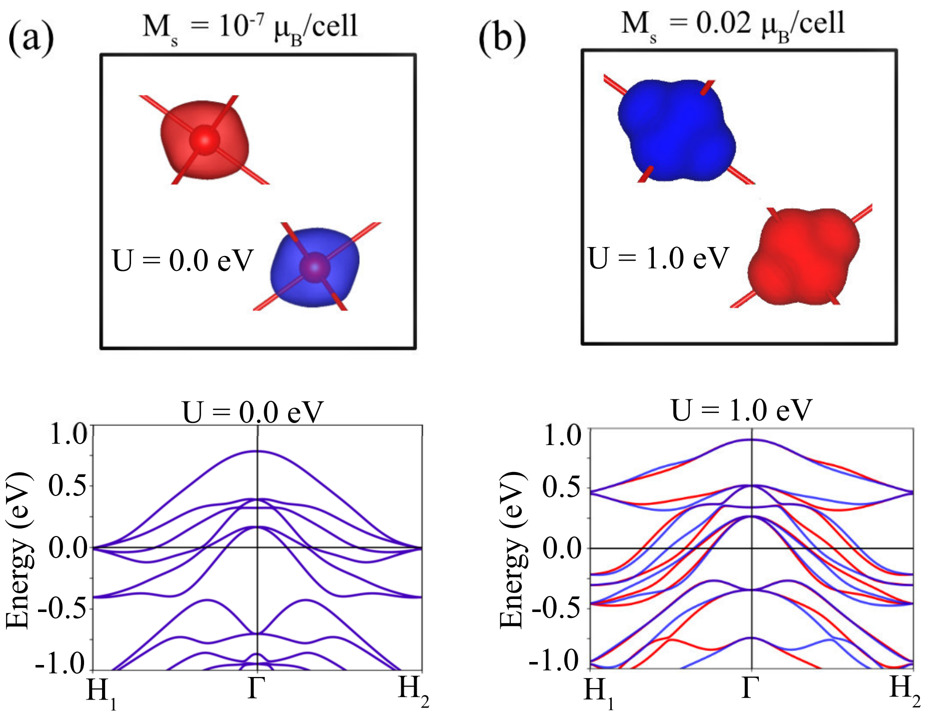

Supplementary figure 13: Real space spin density (S↑ - S↓) and corresponding nonrelativistic band structure of $NiNb_3S_6$ calculated with Hubbard correction $U = 0.0$ eV (a) and $U = 1.0$ eV (b). Red and blue represent spin majority and minority bands. $NiNb_3S_6$ exhibits an altermagnetic phase even without Hubbard U, but the net local spin density around the magnetic sublattices is low. Therefore, all properties of $NiNb_3S_6$ presented in this manuscript are calculated for U = 1.0 eV, unless stated otherwise.

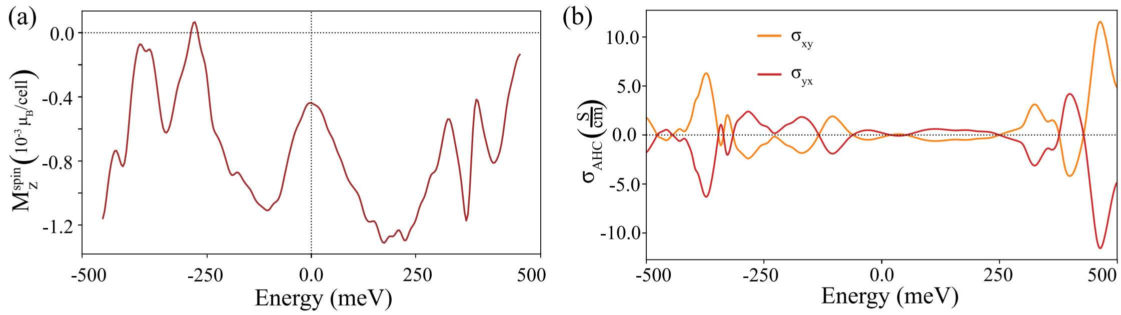

Supplementary figure 14: (a) Spin contribution to magnetization M_z and (b) anomalous Hall effect calculated for $NiNb_3S_6$ with the Néel vector along the x axis. Panel (a) demonstrates the weak ferromagnetism with the net magnetic moment along the z-axis. The spin contributions to magnetization M_x and M_y are of the order of $\sim 10^{-9}$ μ_B/cell, and they are omitted in the plot.

S5: Rashba-Edelstein effect and spin Hall effect in $NiNb_3S_6$

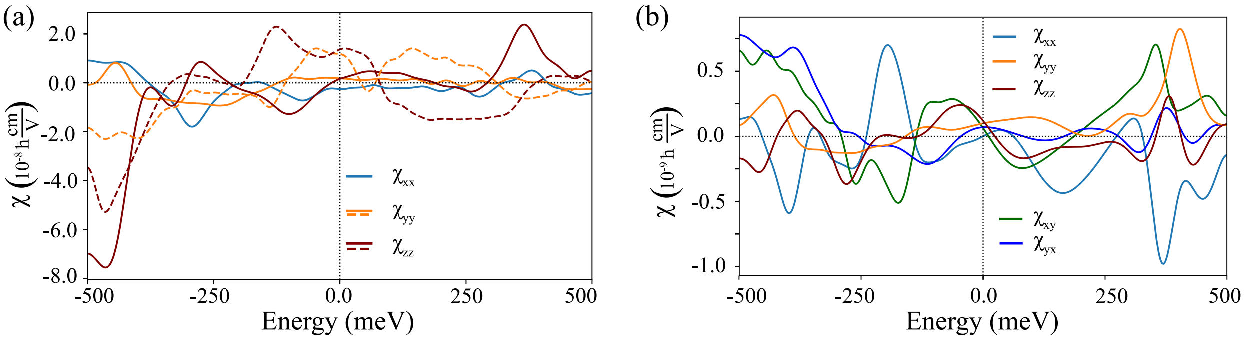

Supplementary figure 15: (a) $\mathcal{T}$-even REE calculated for $NiNb_3S_6$ in nonmagnetic (dashed) and altermagnetic (solid) phases. The plots were evaluated using $\Gamma = 10.0$ meV. For the nonmagnetic phase, $\chi_{xx} = \chi_{yy}$, hence only χ_{yy} is shown. (b) $\mathcal{T}$-odd REE calculated for the altermagnetic phase of $NiNb_3S_6$. Note that the presented scales are different for $\mathcal{T}$-even and $\mathcal{T}$-odd REE with the latter being an order of magnitude lower.

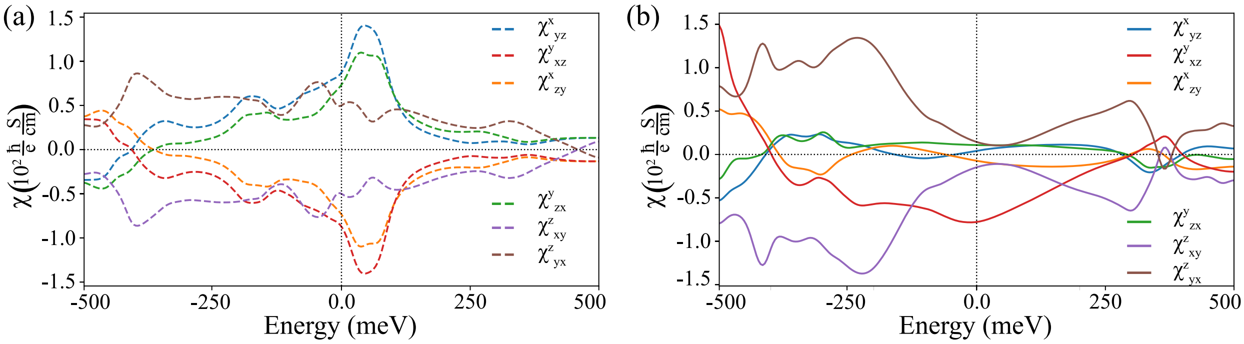

Supplementary figure 16: All allowed tensor components of $\mathcal{T}$-even SHC as a function of chemical potential calculated for nonmagnetic (a) and altermagnetic (b) phase of $NiNb_3S_6$. Note that the relations $\chi^x_{yz} = -\chi^y_{zx}$ and $\chi^y_{zx} = -\chi^x_{zy}$ hold only for the nonmagnetic phase but not for the altermagnetic phase. The relation $\chi^z_{xy} = -\chi^z_{yx}$ holds for both phases. This is in contrast to $Ni_3Ta_3S_6$, where all the relations for $\mathcal{T}$-even SHC hold for both nonmagnetic and altermagnetic phases with the Néel vector along the z-axis.

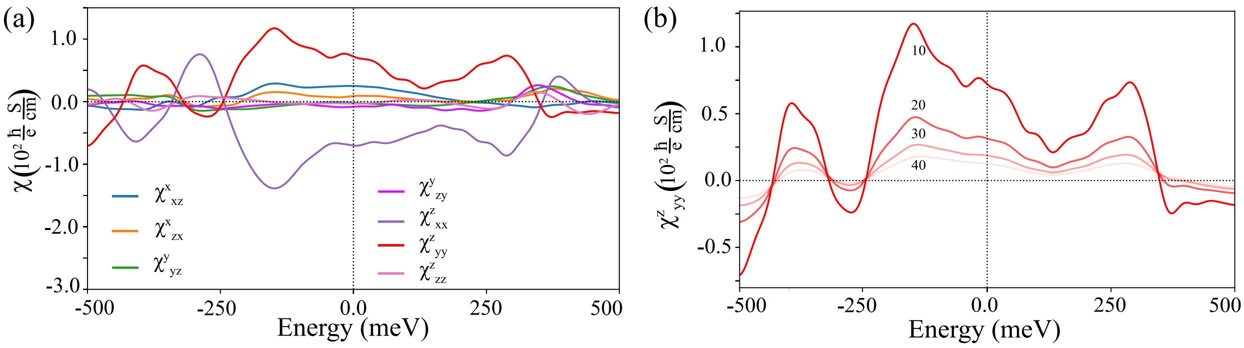

Supplementary figure 17: (a) $\mathcal{T}$-odd SHC calculated as a function of the chemical potential in the altermagnetic $NiNb_3S_6$ with the Néel vector along the x-axis. The plots were evaluated using the parameter Γ = 10.0 meV. (b) $\mathcal{T}$-odd SHC component χ^z_{yy} calculated vs chemical potential for four distinct values of Γ.

S6: Charge-to-spin conversion in $NiTa_3S_6$ and $NiNb_3S_6$ vs reference materials

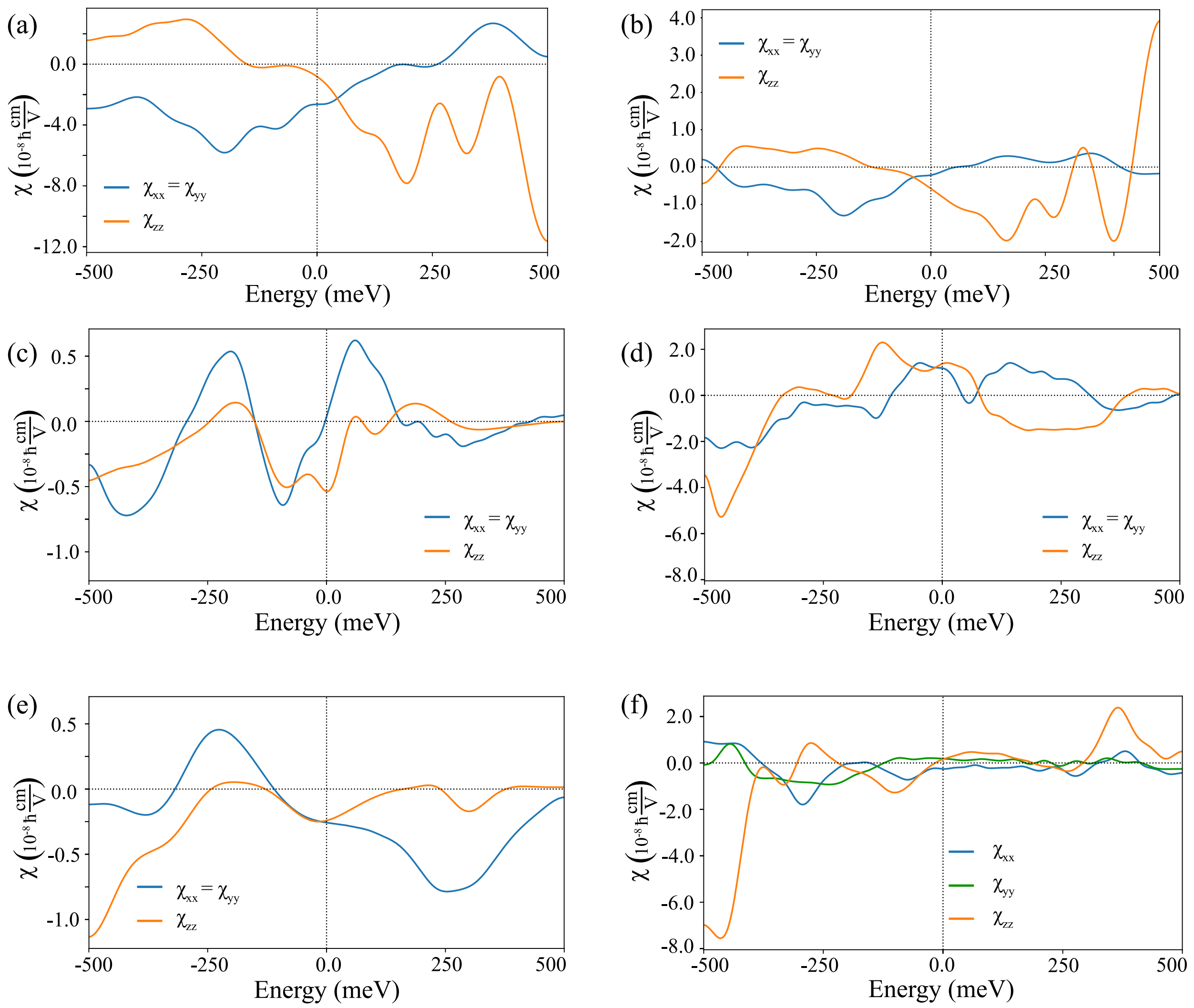

Supplementary figure 18: $\mathcal{T}$-even REE calculated using Kubo's formalism for different chiral materials: Left-handed $TaSi_2$ (a), left-handed $NbSi_2$ (b), nonmagnetic $NiTa_3S_6$ (c), nonmagnetic $NiNb_3S_6$ (d), altermagnetic $NiTa_3S_6$ (e), and altermagnetic $NiNb_3S_6$ (f). The disorder parameter $\Gamma = 10.0$ meV is used for all of them except for $NiTa_3S_6$ for which we set $\Gamma = 30$ meV.
